# Laparoscopic Paraesophageal Hernia Reduction with Two Point Fixation via Ponsky PEG Tube in a Patient in their early 90s

**DOI:** 10.51894/001c.6342

**Published:** 2017-08-24

**Authors:** Catherine Petzinger, John Parmely

**Affiliations:** 1 Beaumont Health General Surgery Resident, PGY 4, Farmington Hills, MI; 2 Beaumont Health Program Director of General Surgery, Farmington Hills, MI

**Keywords:** percutaneous endoscopic gastrostomy (peg) tube, gastropexy, paraesophageal hernia reduction

## Abstract

Paraesphageal hernia (PEH) repairs have been historically controversial due to widely variable clinician opinions. However, there is little research regarding the use of PEH reduction and gastropexy via a percutaneous endoscopic gastrostomy (PEG) tube. Guidelines by the Society of American Gastrointestinal and Endoscopic Surgeons do advise that the use of gastropexy alone is a valid option in patients with high risk of morbidity and mortality, but is associated with high hernia recurrence rates. A male in his early 90s presented with a six-week history of dysphagia, regurgitation and a 30- pound weight loss. Imaging revealed a large PEH and the entire stomach within the thoracic cavity. Despite the patient’s age and significant risk factors, it was determined that he required surgical intervention due to the severity of his symptoms. The safest course of action was reduction of PEH with two-point gastric fixation, rather than a prolonged repair of the hiatus or mesh implant. Due to the patient’s significant surgical risks, it was determined that the safest surgical approach would be laparoscopic reduction with dual gastropexy via PEG tube gastropexy. This approach was quick, without encroachment into the mediastinum and avoided any complications that mesh implantation could have posed. Gastropexy is a relatively simple technique with minimal tissue dissection that is tolerated well in elderly patients or those with decreased cardiac and pulmonary status. Regardless of the surgical PEH approach, there are inherent hernia recurrence rates

## INTRODUCTION

Hiatal hernias are defects in the diaphragm that can allow for aberrant organs to migrate into the chest, and generally categorized into four types. The first type, a Type I hernia, is a sliding hernia in which the gastroesophageal (GE junction) migrates above the diaphragm, accounts for 90% of all hiatal hernias.^1^ Type II-through-IV hernias are categorized as paraesophageal hernias (PEH). Type II hernias have a fixed GE junction below the diaphragm and the superior portion of the stomach (the fundus) is found above the diaphragm. Type III hernias are the most common defects, with both the GE junction and a portion of the stomach displaced above the diaphragm. Type IV hernias entail all the components of Type III defects with another organ (small bowel, colon, etc.) herniated above the diaphragm.^1^

A recent study examining outcomes in “giant” PEH demonstrated that presenting symptoms can be extremely variable.^2^ In this prior study with over 500 patients, the most common presenting symptoms included heartburn (59%), postprandial chest pain (40%), cough (16%), shortness of breath (53%), early satiety (54%), dysphasia (47%), and anemia (37%).^2^ PEH are also more frequently seen in women and the elderly population who may have tolerated their symptoms for years.^3^

**Figure 1:**
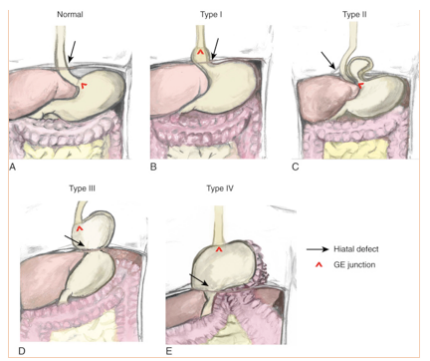
Anatomy of the Esophagus, Gastroesophageal (GE) Junction, and Stomach Displaying Normal Anatomy and the Four Types of Hiatal Hernias **A.** Normal. **B.** Sliding or type I hiatal hernia. **C.** Paraesophageal or type II hiatal hernia. **D.** Type III hiatal hernia **E.** Type IV hiatal hernia (note colon to the left of the herniated gastric fundus. (Illustrations by Lindsay Agema and Christian B. Rodriguez.) Used with permission from Elsevier.

PEH defects are usually asymptomatic, especially Type I. However, a study by Shihvo et al. examined the possible fatal complications associated with PEH.^4^ Major complications included: intrathoracic incarceration of the stomach, gastric volvulus, bleeding, perforation, or decreased pulmonary function.^4^ Based on these risks, this group of earlier authors advocated for repair of PEH in symptomatic patients unless estimated mortality risks of greater than 10%.^4^ Generally, PEHs tend to increase in size over time, and the annual incidence of acute symptoms requiring emergency surgery is estimated to be between 0.7 and 7.0%.^5^ Since a larger-sized hernia can make surgery more technically difficult, patients requiring emergent surgery for complications of PEH have a much higher morbidity and mortality rate than those receiving elective procedures.^5^

Historically, debates concerning different preferred techniques for PEH repairs have endured.^6^ Techniques ranging from open versus laparoscopic to with or without mesh implantation, as well as interventions for symptomatic PEH have evolved greatly over the years.^6^ However, the morbidity and mortality associated with surgical PEH repair has also been decreasing throughout the years.^7^ The latest 2013 Society of American Gastrointestinal and Endoscopic Surgeons (SAGES) guidelines suggest laparoscopic hiatal hernia repair is now as effective as open trans-abdominal repair.^7^ The principles of repair consists of the reduction of the hernia into the abdominal cavity and fixation so that contents cannot rotate within the peritoneal cavity or herniate back into the thorax.

This is usually accomplished with a fundoplication, a fixation of the anterior gastric wall to the abdominal wall, and the repair of the diaphragmatic crura, the tendons of the diaphragm that surround the opening for the esophagus. This crura defect has historically been closed with mesh. Plication of the stomach to the anterior abdominal wall by temporary gastrostomy tube could also protect against subsequent gastric volvulus. However, there is little research or evidence to support the use of gastropexy alone to manage PEH. The most recent SAGES guidelines advise that gastropexy alone is a valid option in patients with high risk of morbidity and mortality.^6^

At time of operation, an older age, lower body mass index, and a larger preoperative hernia are significantly associated with an increased rate of postoperative morbidity.^7^ Fundoplication and crural repair can be lengthy and difficult especially in patients who have very large PEH defects. Although some surgeons use mesh to close such defects, there are intrinsic complications with mesh implantation. Complications include mesh migration, infection, dysphagia and erosion of mesh into surrounding structures. Even though gastropexy alone comes with an increased risk of recurrent herniation, it remains a viable surgical option for medically high-risk patients.

### Case Report

This case report concerns a male in his early 90s with a pertinent past medical history of smoking and chronic obstructive pulmonary disease (COPD), right bundle branch block arrhythmia, hypertension and hypothyroidism. He presented to his primary care provider’s office with a six-week history of complaints of dysphagia (difficulty swallowing) with regurgitation of food as well as reported 30-pound weight loss. He denied any previous gastroesophageal reflux symptoms. An outpatient CT scan of his abdomen revealed a large PEH with the entire stomach within an intra-thoracic PEH defect. The patient presented to the authors’ surgical office to discuss options.

**Figure 2:**
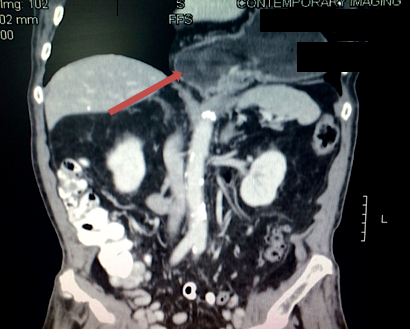
CT Scan of Abdomen Showing the Stomach Completely Above the Diaphragm.

Despite the patient’s age and significant risk factors (i.e., smoking, hypertension, large hernia, poor nutritional status and low body mass index), the authors determined that the patient needed surgical intervention due to the severity of his acute obstructive symptoms. They concluded that the safest course of action was reduction of PEH with two-point gastric fixation with percutaneous endoscopic gastrostomy (PEG) tubes, rather than subject the patient to a longer PEH repair with reinforcement mesh or fundoplication. The authors also had concerns about the patients’ nutritional status due to his significant weight-loss and its effects on his postoperative healing, making this shorter less invasive alternative ideal for the patient.

## Surgical Intervention and Hospital Course

Intraoperatively, the patient was found to have a large hiatal defect with the majority of the stomach within the paraesophageal defect and a gastric volvulus. The stomach was easily reduced into the abdominal cavity without any dissection into the mediastinum. The gastric volvulus was also reduced. Subsequently, the gastric outlet was noted to be patent on evaluation with a gastroscope. There was also no evidence of gastritis or esophagitis on EGD. Two Ponsky PEG tubes were then placed using endoscopic/percutaneous technique with laparoscopic visualization for positioning of the PEG tubes. The PEG tubes were placed at the proximal and distal stomach, performing a pexy (fixation) of the anterior wall of the stomach to the abdominal wall.

**Figure 3:**
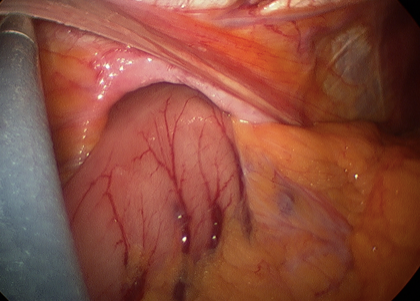
Laparoscopic View of Paraesophageal Hernia and Crural Defect

**Figure 4:**
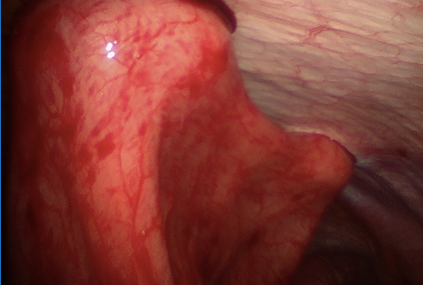
Laparoscopic View After Reduction and Two-Point Ponsky Fixation to the Anterior Abdominal Wall.

Postoperatively, the patient was admitted to the hospital for monitoring and postoperative care. Although he was at first hesitant to resume eating, he resumed a regular diet after some encouragement without signs of dysphagia. An Upper GI Study was performed on postoperative day #1 showing the cardia of the stomach in the left hemithorax. However, the remainder of the stomach was within the abdomen and there was no gastric outlet obstruction or volvulus. The patient was discharged home on postoperative day #2 without any signs of dysphagia. The patient was followed closely on an outpatient basis for one postoperative month and continued to tolerate a regular diet, gaining weight without enteral supplementation. After the post-op month, both Ponsky PEG tubes were removed without any recurrence of symptoms.

## DISCUSSION

Paraesophageal hernias, Types II-IV should be repaired in a timely manner due to the significant comorbidities associated with these defects. These hernias will continue to increase in size over time, making the non-emergent repair of these hernias necessary in younger patients. Earlier repairs result in fewer intra-thoracic adhesions, making the dissection of the hernia from the thoracic cavity less complicated. Dilation of the hiatus and stretching of the cura also increases with time, which can also make surgical repair more difficult. The approach and type of surgery performed should be chosen based on the patient factors and fit the patients’ surgical profile. Elective repairs avoid future complications and improve patients’ function.^3^

Traditional management of PEH calls for a lengthy and risky surgical approach that is not always appropriate for patients with advanced age and multiple co-morbidities. In a 2010 article by Luketich et al., 662 patients who underwent laparoscopic giant PEH repairs were followed for 10 years.^8^ These patients underwent screening esophagrams which showed hiatal hernia recurrence of up to 15.7% although only 3.2% had return of their symptoms. In this 2010 study, those patients, who underwent complete dissection of the intra-thoracic hernia sac, crural repair and mesh application still had a 15.7% recurrence rate.^8^ In a similar study from Antonoff and colleagues, their group of patients underwent similar steps for their PEH repairs with morbidity rates of 8.2% and recurrence rates of 5.5%.^9^ These authors attributed their success rates to observing a minimally invasive approach while still using the fundamentals of open repair.^9^

Some surgeons are proponents of adding a wrap of the fundus of the stomach after the hernia is reduced and mesh is fixed to prevent the occurrence of GERD. One German randomized controlled pilot study with 40 patients compared Laparoscopic mesh-augmented hiatoplasty with simple cardiophrenicopexy (LMAH-C) versus Laparoscopic mesh-augmented hiatoplasty with fundoplication.^10^ Their data showed that recurrence rates were similar between the two (33% and 21%, respectively). However they proposed that fundoplication be added to all repairs due to the risk of new onset GERD that occurred in a significant amount of patients who underwent LMAH-C (53%) in comparison to those who underwent LMAH-F (17%). Moreover, adding a fundoplication also added half an hour to surgery (Mean 153 minutes; range 90-250 minutes) in comparison to LMAH–C (Mean 124 minutes: range 85-210 minutes).^10^

Different surgical approaches, techniques and modalities for PEH repair add an increased amount of time under anesthesia for patients who are elderly and frail, increasing their morbidity and possible mortality risks. Mortality rates after emergent PEH repair are estimated at 5.4-8% and 0.8-1.4% after elective PEH, and frail patients have higher mortality risks.^5^

Our approach in this case was not only unique in the use of dual PEG tube insertion to prevent gastric volvulus, but also allowed a chronically ill patient to undergo surgical intervention for which he otherwise would not have been a candidate. Due to the significant risks attributed with this patient in their early 90s, the authors determined that the safest approach to his large PEH would be laparoscopic reduction of the PEH with gastropexy. This approach was relatively quick (76 minutes), without encroachment into the mediastinum and avoided any complications mesh implantation could pose. SAGES guidelines do report that morbidity and mortality of gastropexy is significantly lower than other more invasive PEH repairs.^7^ However, gastropexy alone has not demonstrated the same efficacy as formal repair of a PEH and should be reserved for patients with significant comorbidities.^7^

Gastropexy is a relatively simple technique with minimal tissue dissection that is generally well tolerated in the elderly population and those with decreased cardiac and pulmonary status.^11^ Regardless of the approach to PEH repair, there are inherent chances of recurrence. In a study from Daigle and associates, laparoscopic PEH repairs using a modified Boerema anterior gastropexy (i.e., fixation to the abdominal wall with nonabsorbable sutures putting the esophagus under some tension to form an “angle of His”) was completed without fundoplication at multiple centers.^12^ Out of 101 patients, 70% had no postoperative reflux with recurrence rates of 16.8% when followed on endoscopy or barium swallow evaluation.^12^ Surgical intervention and approach should therefore be tailored to the patient, with risks and benefits carefully weighed. In this case, the patient tolerated dual gastropexy alone with complete resolution of symptoms without the increased accompanying risks of crural repair and mesh implantation.

### Conflict of Interest

The authors declare no conflict of interest.
